# Some aspects of an induced electric dipole moment in rotating and non-rotating frames

**DOI:** 10.1098/rsos.170541

**Published:** 2017-06-28

**Authors:** Abinael B. Oliveira, Knut Bakke

**Affiliations:** Departamento de Física, Universidade Federal da Paraíba, Caixa Postal 5008, 58051-900, João Pessoa-PB, Brazil

**Keywords:** rotating effects, induced electric dipole moment, Landau quantization, Kratzer potential, linear scalar potential

## Abstract

Quantum effects on a neutral particle (atom or molecule) with an induced electric dipole moment are investigated when it is subject to the Kratzer potential and a scalar potential proportional to the radial distance. In addition, this neutral is placed in a region with electric and magnetic fields. This system is analysed in both non-rotating and rotating reference frames. Then, it is shown that bound state solutions to the Schrödinger equation can be achieved and, in the search for polynomial solutions to the radial wave function, a restriction on the values of the cyclotron frequency is analysed in both reference frames.

## 1. Introduction

In the literature, crossed electric and magnetic fields have attracted interest in studies of the hydrogen atom [1–6], large electric dipole moments [7], atoms and molecules in strong magnetic field [8–10], geometric quantum phases [11,12] and the quasi-Landau behaviour in atomic systems [13,14]. In [15], it is shown that crossed electric and magnetic fields can give rise to an analogue of the Landau quantization [16] for an atom with an induced electric dipole moment. Recently, this interaction of the induced electric dipole moment of an atom with a uniform effective magnetic field produced by crossed electric and magnetic fields has been investigated in quantum rings [17], subject to scalar potentials [18,19] and in rotating reference frames [20,21]. Other interesting effects that arise from quantum fluctuations in the QED vacuum have been reported in [22,23]. The focus of this work is on the quantum effects on an atom (molecule) with an induced electric dipole moment subject to the Kratzer potential [24–28] and a scalar potential proportional to the radial distance in a region with crossed electric and magnetic fields. We deal with this system in both non-rotating and rotating frames. Particular interest in rotating systems arise from studies of geometric quantum phases [29–39], spintronics [40–42], quantum rings [43,44] scalar bosons [45], DKP equation [46] and electroweak interactions [47]. Therefore, the present study fills a lack in the studies of neutral particles with no permanent electric dipole moment that interacts with external fields.

The structure of this paper is: in §2, we introduce the quantum description of a moving neutral particle (molecule or atom) with an induced electric dipole moment in a region with electric and magnetic fields; thus, we consider the field configuration proposed in [15] that gives rise to the analogue of the Landau quantization and analyse this system subject to the Kratzer potential [24–26] and a scalar potential proportional to the radial distance; in §3, we investigate the quantum effects on the system described in the previous section by considering a rotating reference frame; in §4, we present our conclusions.

## 2. Non-rotating frame

Let us begin this section by reviewing the quantum description of an atom or molecule with an induced electric dipole moment that moves with a velocity *v*≪*c* and interacts with external fields. As shown in [11,15,18,19,21], the Hamiltonian operator of the system is given by (with ℏ=c=1):

2.1 $$H_{0}=\frac{1}{2m}{\left(\hat{p}+\alpha \;\text{E}\times \text{B}\right)}^{2}-\frac{\alpha }{2}E^{2}+V,$$

where *m* is the mass of the particle, *α* is is the dielectric polarizability, V is the potential energy, $\hat{p}=-i\nabla$ is the momentum operator (vector operator) and the vectors ***E*** and ***B*** are the electric and magnetic fields in the laboratory frame, respectively. According to Wei *et al.* [12], the term *αE*^2^ given in equation (2.1) is very small compared with the kinetic energy of the atoms, therefore, we can neglect it without loss of generality from now on.

The focus of this section is on the quantum effects on an atom (molecule) with an induced electric dipole moment in a region with a uniform effective magnetic field, which is subject to the Kratzer potential [24–26] and a scalar potential proportional to the radial distance. In particular, this uniform effective magnetic field is defined as ***B***_eff_=**∇**×(***E***×***B***), where the magnetic and electric fields in the laboratory frame are [15]

2.2 $$B_{z}=B_{0}\;\mathrm{and}\;E_{r}=\frac{\lambda r}{2},$$

where *B*_0_ is a constant and *λ* is a constant related to the uniform volume charge density. As observed in [15], the interaction of the induced electric dipole moment of the atom with the magnetic and electric fields given in equation (2.2) gives rise to a discrete spectrum of energy which is known as an analogue of the Landau quantization [16]. Recently, quantum effects on this Landau-type system have been investigated in a quantum ring [17], under the influence of a Coulomb-type potential [18], a linear confining potential [19] and the Kratzer potential in a rotating frame [21]. Besides, in order to investigate the influence of the Kratzer potential [24–26] and a scalar potential proportional to the radial distance, then, we write the potential energy V as follows:

2.3 $$V=br-\frac{2Da}{r}+\frac{Da^{2}}{r^{2}}.$$

The first term in the above equation corresponds to the the scalar potential proportional to the radial distance, where *b* is a constant. The second term corresponds to the Kratzer potential, where *D* and *a* are constants. It has attracted great interest in studies of molecules [48–50]. Thereby, after substituting equations (2.2) and (2.3) into equation (2.1), the time-independent Schrödinger equation becomes

2.4 $$E\Psi =-\frac{1}{2m}\nabla^{2}\Psi +i\frac{\alpha \lambda B_{0}}{2m}\frac{\partial \Psi }{\partial \varphi }+\frac{\alpha^{2}\lambda^{2}B_{0}^{2}}{8m}r^{2}\Psi -\frac{2Da}{r}\Psi +\frac{Da^{2}}{r^{2}}\Psi +br\Psi ,$$

where ∇^2^ is the Laplacian in cylindrical coordinates. Let us take *Ψ*(*r*,*φ*,*z*)=*Φ*(*φ*)*Z*(*z*)*F*(*r*); thus, we have that *Φ*(*φ*)=*e*^i*νφ*^, where *ν*=0,±1,±2,… and *Z*(*z*)=*e*^i*kz*^, where *k* is a constant. Then, by defining the parameter *ω*=*αλB*_0_/*m* as the cyclotron frequency associated with the Landau quantization for an atom with an induced electric dipole moment [15], let us substitute *Ψ*(*r*,*φ*,*z*) into equation (2.4) and perform a change of variables given by

2.5 $$y=\sqrt{\frac{m\omega }{2}}r.$$

In this way, we obtain the following second order differential equation:

2.6 $$F^{\prime \prime }+\frac{1}{y}F^{\prime }-\frac{\tau^{2}}{y^{2}}F-y^{2}F+\frac{\mu }{y}F-\theta yF+\chi F=0,$$

where *τ*^2^=*ν*^2^+2*mDa*^2^, $\mu =4mDa/\sqrt{m\omega /2}$, *θ*=2*mb*/(*mω*/2)^3/2^ and $\chi =(2/m\omega )[2mE-k^{2}+m\omega \nu ]$. It is well known that the analysis of the asymptotic behaviour of equation (2.6) determines the form of the function *F*(*y*), therefore, this function can be written in terms of an unknown function *H*(*y*) as

2.7 $$F(y)=e^{-y^{2}/2-\theta y/2}y^{|\tau |}H(y).$$

By substituting equation (2.7) into equation (2.6), we obtain that *H*(*y*) is the solution to the following equation:

2.8 $$H^{\prime \prime }+\left[\frac{2|\tau |+1}{y}-\theta -2y\right]H^{\prime }+\left[\chi +\frac{\theta^{2}}{4}-2|\tau |-2+\frac{2\mu -\theta (2|\tau |+1)}{2y}\right]H=0,$$

which is the biconfluent Heun equation [51], then, *H*(*y*)=*H*_*B*_(2|*τ*|,*θ*,*χ*+*θ*^2^/4,−2*μ*;*y*) is the biconfluent Heun function.

We wish the function *F*(*y*) goes to zero when y→∞ and y→0, therefore, let us use the Frobenius method [52]. In this method, we first write the biconfluent Heun function as a power series around the origin, $H(y)=\sum_{i=0}^{\infty }b_{i}y^{i}$, and then, we search for polynomial solutions to the biconfluent Heun equation (2.8). By substituting this series in equation (2.8) we obtain the relation

2.9 $$b_{1}=\left[\theta -\frac{2\mu }{2|\tau |+1}\right]b_{0}$$

and the recurrence relation:

2.10 $$b_{i+2}=\frac{\theta (i+1)-2\mu +\theta (2|\tau |+1)}{\left(i+2)(i+2+2|\tau |\right)}b_{i+1}+\frac{2i-\chi -\theta^{2}/4+2+2|\tau |}{\left(i+2)(i+2+2|\tau |\right)}b_{i}.$$

From the recurrence relation above, we have that the biconfluent Heun series becomes a polynomial of degree *n* by imposing that [51]

2.11 $$\chi +\frac{\theta^{2}}{4}-2|\tau |-2=2n;\;b_{n+1}=0,$$

for *n*=1,2,3,… Hence, these two conditions must be analysed in order to achieve a polynomial solution. From the condition *χ*+*θ*^2^/4−2|*τ*|−2=2*n*, we obtain

2.12 $$E_{n,\nu }=\frac{1}{2}\omega [n+|\tau |-\nu +1]-\frac{2b^{2}}{m\omega^{2}}+\frac{k^{2}}{2m},$$

which yields the energy levels of a neutral particle with an induced electric dipole moment in a region with crossed electric and magnetic fields under the influence of scalar potentials. Observe that the field configuration of electric and magnetic field given in equation (2.2) gives rise to a uniform effective magnetic field in the *z*-direction that yields an analogue of the Landau quantization as pointed out in [15]. By comparing the energy levels (2.12) with the analogue of the Landau levels in [15], we have that the presence of the Kratzer potential and the scalar potential proportional to the radial distance modifies the energy levels and breaks the degeneracy of the Landau-type levels.

On the other hand, our search for polynomial solutions to the biconfluent Heun series will be completed when we analyse the condition *b*_*n*+1_=0 given in equation (2.11). For this purpose, let us consider *b*_0_=1. Then, let us construct a polynomial of first degree. For *n*=1, we obtain *b*_2_=0 from *b*_*n*+1_=0. In this way, we obtain

2.13 $$\omega_{1,\nu }^{3}-\frac{64mD^{2}a^{2}}{2|\tau |+1}\omega_{1,\nu }^{2}+\frac{32b\;Da(4|\tau |+3)}{2|\tau |+1}\omega_{1,\nu }-\frac{32b^{2}(|\tau |+1)}{m}=0,$$

which is a third degree algebraic equation. It means that, in order to achieve a polynomial of first degree to *H*(*y*), we have that not all values of the cyclotron frequency are permitted, but only which are determined by the third degree algebraic equation (2.13) [53]. Despite having at least one real solution to the third degree algebraic equation (2.13), we do not write this solution because it is very long. Besides, for each energy level *n* of the system, we can have a different expression that determines the possible values of the cyclotron frequency. For this reason, we have labelled *ω*=*ω*_*n*,*l*_ in equation (2.13) and rewrite the energy levels (2.12) in the form:

2.14 $$E_{n,\nu }=\frac{1}{2}\omega_{n,\nu }[n+|\tau |-\nu +1]-\frac{2b^{2}}{m\omega_{n,\nu }^{2}}+\frac{k^{2}}{2m},$$

which are the energy levels of a neutral particle with an induced electric dipole moment in a region with a uniform effective magnetic field, where there exists the influence of the Kratzer potential and a scalar potential proportional to the radial distance.

## 3. Rotating frame

In this section, we consider a rotating frame where the system discussed in the previous section is in a reference frame that rotates with a constant angular velocity $\Omega =\Omega \hat{z}$. By following [17,36,37,54–57], the time-independent Schrödinger equation in the rotating frame is given by

3.1 $$H_{0}\Psi -\Omega \cdot \hat{L}\Psi =E\Psi ,$$

where $H_{0}$ is given in equation (2.1), i.e. it is to the Hamiltonian operator in the absence of rotation, and $\hat{L}$ is the angular momentum operator. In the present case, the angular momentum operator is given by $\hat{L}=\text{r}\times (\hat{p}+\alpha \text{E}\times \text{B})$, where $\text{r}=r\hat{r}$ in a two-dimensional system. Thereby, the time-independent Schrödinger equation (3.1) becomes

3.2 $$\begin{array}{rl} E\Psi & =-\frac{1}{2m}\nabla^{2}\Psi +i\frac{\alpha \lambda B_{0}}{2m}\frac{\partial \Psi }{\partial \varphi }+\frac{\alpha^{2}\lambda^{2}B_{0}^{2}}{8m}r^{2}\Psi \\ & \;+i\Omega \frac{\partial \Psi }{\partial \varphi }+\frac{\Omega \alpha \lambda B_{0}}{2}r^{2}\Psi -\frac{2Da}{r}\Psi +\frac{Da^{2}}{r^{2}}\Psi +br\Psi . \end{array}$$

We can go further by taking *Ψ*(*r*,*φ*,*z*)=*e*^i*lφ*+i*kz*^ *G*(*r*) in equation (3.2), however, let us define a new parameter *ϖ* through the relation:

3.3 $$\varpi^{2}=\omega^{2}+4\Omega \omega ,$$

where *ω*=*αλB*_0_/*m* is the cyclotron frequency as established in [15]. With this new parameter, let us perform a new change of variables:

3.4 $$x=\sqrt{\frac{m\varpi }{2}}\rho$$

and thus, we obtain

3.5 $$G^{\prime \prime }+\frac{1}{x}G^{\prime }-\frac{\tau^{2}}{x^{2}}G-x^{2}G+\frac{\bar{\mu }}{x}G-\bar{\theta }xG+\bar{\chi }G=0,$$

where *τ*^2^=*l*^2^+2*mDa*^2^, $\bar{\mu }=4mDa/\sqrt{m\varpi /2}$, $\bar{\theta }=2mb/{\left(m\varpi /2\right)}^{3/2}$ and $\bar{\chi }=(2/m\varpi )[2mE-k^{2}+m\omega l+2ml\Omega ]$.

The behaviour of the function *G*(*x*) at x→∞ and x→0 allows us to write the function *G*(*x*) in terms of an unknown function $\bar{H}(x)$ as

3.6 $$G\left(x\right)=e^{-x^{2}/2-\bar{\theta }x/2}x^{|\tau |}\bar{H}(x)$$

and then, after substituting equation (3.6) into equation (3.5), we obtain

3.7 $${\bar{H}}^{\prime \prime }+\left[\frac{2|\tau |+1}{x}-\bar{\theta }-2x\right]{\bar{H}}^{\prime }+\left[\bar{\chi }+\frac{{\bar{\theta }}^{2}}{4}-2|\tau |-2+\frac{2\bar{\mu }-\bar{\theta }\left(2|\tau |+1\right)}{2x}\right]\bar{H}=0,$$

which is also the biconfluent Heun equation [51] and $\bar{H}(x)=H_{B}(2|\tau |$, $\bar{\theta },\chi +{\bar{\theta }}^{2}/4,-2\bar{\mu };x)$ is the biconfluent Heun function.

By following the steps from equation (2.8) to equation (2.11), we obtain the relations:

3.8 $$b_{1}=\left[\bar{\theta }-\frac{2\bar{\mu }}{2|\tau |+1}\right]b_{0}$$

and

3.9 $$b_{i+2}=\frac{\bar{\theta }(i+1)-2\bar{\mu }+\bar{\theta }(2|\tau |+1)}{\left(i+2)(i+2+2|\tau |\right)}b_{i+1}+\frac{2i-\bar{\chi }-{\bar{\theta }}^{2}/4+2+2|\tau |}{\left(i+2)(i+2+2|\tau |\right)}b_{i}.$$

Besides, in this case we have that the biconfluent Heun series becomes a polynomial of degree *n* by imposing that [51]

3.10 $$\bar{\chi }+\frac{{\bar{\theta }}^{2}}{4}-2|\tau |-2=2n;\;b_{n+1}=0,$$

for *n*=1,2,3,…. Then, from the condition $\bar{\chi }+({\bar{\theta }}^{2}/4)-2|\tau |-2=2n$, we obtain

3.11 $$E_{n,l}=\frac{1}{2}\sqrt{\omega^{2}+4\Omega \omega }[n+|\tau |+1]-\frac{1}{2}\omega l-\frac{2\eta^{2}}{m(\omega^{2}+4\Omega \omega )}+\frac{k^{2}}{2m}-\Omega l,$$

where *n*=1,2,3,… and *l*=0,±1,±2,…. Hence, equation (3.11) corresponds to the energy levels of the system in a rotating reference frame. In contrast with the analogue of the Landau levels [15], the change in the energy levels and in the degeneracy of them are owing to the effects of rotation, the presence of the Kratzer potential and the scalar potential proportional do the radial distance. Furthermore, the effects of rotation give rise to the coupling to the angular momentum quantum number and the angular velocity, which is known as the Page-Werner *et al.* term [58–60]. Note that by taking Ω→0, we recover the energy levels (2.12).

By following the same analysis of the condition *b*_*n*+1_=0 made in equation (2.13), we obtain that possible values of the cyclotron frequency associated with the lowest energy state (*n*=1) are determined by

3.12 $$\begin{array}{rl} & {\left(\omega_{1,l}^{2}+4\Omega \omega_{1,l}\right)}^{3/2}-\frac{64mD^{2}a^{2}}{2|\tau |+1}(\omega_{1,l}^{2}+4\Omega \omega_{1,l})+\frac{32\eta Da(4|\tau |+3)}{2|\tau |+1}{\left(\omega_{1,l}^{2}+4\Omega \omega_{1,l}\right)}^{1/2}\\ & \;-\frac{32\eta^{2}(|\tau |+1)}{m}=0. \end{array}$$

Hence, we can see that the possible values of the cyclotron frequency are determined by the quantum numbers of the system {*n*,*l*} and the parameters that characterizes the rotation, the Kratzer potential and the scalar potential proportional to the radial distance. Again, we can label *ω*=*ω*_*n*,*l*_ and rewrite the energy levels (3.11) in the form:

3.13 $$E_{n,l}=\frac{1}{2}\sqrt{\omega_{n,l}^{2}+4\Omega \omega_{n,l}}[n+|\tau |+1]-\frac{1}{2}l\omega_{n,l}-\frac{2\eta^{2}}{m(\omega_{n,l}^{2}+4\Omega \omega_{n,l})}+\frac{k^{2}}{2m}-\Omega l,$$

which is the general expression of the spectrum of energy of a neutral particle with an induced electric dipole moment in a rotating reference frame under the influence of a region with a uniform effective magnetic field, the Kratzer potential and a scalar potential proportional to the radial distance.

## 4. Conclusion

We have investigated quantum effects on a neutral particle with no permanent electric dipole moment in both non-rotating and rotating reference frames when this quantum particle is subject to scalar potentials. We have also considered a field configuration of electric and magnetic fields that gives rise to a uniform effective magnetic field perpendicular to the *xy*-plane, and then, we have searched for bound state solutions to the Schrödinger equation.

In the first case investigated, we have considered a non-rotating reference frame, and thus, obtained the general expression for the energy levels of the system, where we have seen that the presence of the Kratzer potential and the scalar potential proportional to the radial distance modifies the energy levels and breaks the degeneracy of the Landau-type levels [15]. However, in search of polynomial solutions to the biconfluent Heun equation, we have seen that there is a restriction on the possible values of the cyclotron frequency characterized by the dependence of the cyclotron frequency on the quantum numbers of the system and the parameters associated with the Kratzer potential and the scalar potential proportional to the radial distance. As an example, we have seen that the possible values of the cyclotron frequency associated with the lowest energy state of the system are determined by a third-degree algebraic equation.

In the second case investigated, it is considered a rotating reference frame, hence, a general expression for the energy levels is obtained, where we have seen that the change in the energy levels and in the degeneracy in contrast to the Landau-type levels [15] are owing to the effects of rotation and the presence of scalar potentials. Furthermore, we have obtained a contribution to the energy levels given by the coupling to the angular momentum quantum number and the angular velocity, which is called the Page-Werner *et al.* term [58–60]. Furthermore, in search of polynomial solutions to the biconfluent Heun equation, we have also observed a restriction on the possible values of the cyclotron frequency, where each possible value is determined by the angular velocity of the rotating frame, the parameters associated with the scalar potentials and the quantum numbers of the system. In particular, we have seen that the possible values of the cyclotron frequency associated with the lowest energy state of the system differs from the case of the non-rotating reference frame and they are determined by equation (3.12).

## Data accessibility

This work does not have any experimental data.
